# Cytolethal Distending Toxin in Isolates of *Aggregatibacter actinomycetemcomitans* from Ghanaian Adolescents and Association with Serotype and Disease Progression

**DOI:** 10.1371/journal.pone.0065781

**Published:** 2013-06-14

**Authors:** Carola Höglund Åberg, Georgios Antonoglou, Dorte Haubek, Francis Kwamin, Rolf Claesson, Anders Johansson

**Affiliations:** 1 Division of Molecular Periodontology, Department of Odontology, Faculty of Medicine, Umeå University, Umeå, Sweden; 2 Section for Pediatric Dentistry, Department of Dentistry, Health, Aarhus University, Aarhus, Denmark; 3 Dental School University of Ghana, Accra, Ghana; 4 Division of Oral Microbiology, Department of Odontology, Faculty of Medicine, Umeå University, Umeå, Sweden; University of Malaya, Malaysia

## Abstract

**Background and Objectives:**

The cytolethal distending toxin **(**Cdt) is a highly conserved exotoxin that are produced by a number of Gram negative bacteria, including *Aggregatibacter actinomycetemcomitans,* and affects mammalian cells by inhibiting cell division and causing apoptosis. A complete *cdt*-operon is present in the majority of *A. actinomycetemcomitans*, but the proportion of isolates that lack *cdt*-encoding genes (*A, B* and *C*) varies according to the population studied. The objectives of this study were to examine serotype, *Cdt*-genotype, and Cdt-activity in isolates of *A. actinomycetemcomitans* collected from an adolescent West African population and to examine the association between the carrier status of *A. actinomycetemcomitans* and the progression of attachment loss (AL).

**Materials and Methods:**

A total of 249 *A*. *actinomycetemcomitans* isolates from 200 Ghanaian adolescents were examined for serotype and *cdt-*genotype by PCR. The activity of the Cdt-toxin was examined by DNA-staining of exposed cultured cells and documented with flow cytometry. The periodontal status of the participants was examined at baseline and at a two-year follow-up.

**Results:**

Presence of all three *cdt*-encoding genes was detected in 79% of the examined *A*. *actinomycetemcomitans* isolates. All these isolates showed a substantial Cdt-activity. The two different *cdt*-genotypes (with and without presence of all three *cdt-*encoding genes) showed a serotype-dependent distribution pattern. Presence of *A. actinomycetemcomitans* was significantly associated with progression of AL (OR = 5.126; 95% CI = [2.994–8.779], *p*<0.001).

**Conclusion:**

*A. actinomycetemcomitans* isolated from the Ghanaian adolescents showed a distribution of serotype and *cdt*-genotype in line with results based on other previously studied populations. Presence of *A. actinomycetemcomitans* was significantly associated with disease progression, in particular the b serotype, whereas the association with disease progression was not particularly related to *cdt-*genotype, and Cdt-activity.

## Introduction

Colonization of bacteria that adhere to and develop biofilm on teeth and the surrounding tissues are involved in the pathogenesis of periodontitis [1], [2]. Pathogens located in the subgingival biofilm release components that induce processes in the host response that can result in loss of the tooth supporting tissues [3].

More than 700 different bacterial species can be detected in samples from the subgingival plaque biofilm and other sites of the oral cavity [4]. The majority of these species can be detected in samples from both healthy and periodontally diseased subjects. Some of these species are detected in increased numbers or proportions in plaque samples from diseased subjects and have the capacity to express unique virulence factors associated with pathogenic mechanisms [3].

By use of molecular genetic tools, a biodiversity has been demonstrated in the oral microbiota. Among the periodontal pathogens, *Aggregatibacter actinomycetemcomitans* is often found in high numbers and proportions in plaque samples from subjects with periodontitis, specifically of its localized aggressive form (LAP) [5]. This Gram-negative capnophilic coccobacillus is genetically heterogeneous and comprises distinct clonal lineages that may have different virulence potentials [6]–[8].

*A. actinomycetemcomitans* possesses a number of important virulence factors [9]. One of them is a leukotoxin, which is a large pore-forming protein of the RTX (repeats in toxin) family that specifically activates and lyses human leukocytes and induces a substantial release of IL-1β from macrophages [10]. A specific clone (JP2) of *A. actinomycetemcomitans* has a significantly enhanced expression of the leukotoxin and is strongly associated with LAP in adolescents of African descent [11], [12]. The cytolethal distending toxin (Cdt), also an exotoxin, blocks cell cycle progression in all types of host cells [13], [14].

Seven serotypes (a–g) have been identified among *A. actinomycetemcomitans* isolates, representing distinct clonal lineages [15]–[18]. There is convincing evidence of differences in serotype distribution related to geography and/or ethnic groups [6], [11], [12]. *A. actinomycetemcomitans* isolates from individuals in European countries are usually represented by almost equal proportions of a, b, and c serotypes [19]–[21]. In contrast, several studies showed a clear predominance of serotype c in populations living in Asia and America [22]–[24]. The serotype distribution in the West African population is unknown. The high genetic diversity in isolates of the same serotype of *A. actinomycetemcomitans* [7], [8] indicates that the serotype might be a weak marker for the pathogenic potential of an isolate. It has been suggested that serotype b of *A. actinomycetemcomitans* has a higher pathogenic potential than the other serotypes [25], [26].

Cdt is a highly conserved exotoxin produced by a number of Gram negative bacteria. It affects mammalian cells by inhibiting cell division and causing apoptosis [27]. The active holo-toxin is a heterotrimeric complex of CdtA, CdtB, and CdtC. CdtA and CdtC are necessary for the secretion of the toxin, while CdtB is responsible for the biologic activity [13]. CdtB has a sequence homology with mammalian DNase I, indicating a critical role for nuclease activity in host parasite interactions [28]. The Cdt was firstly discovered in *A. actinomycetemcomitans* by Sugai and co-workers in 1998 [29]. Apart from blocking cell cycle progression, Cdt also induces expression of the receptor activator of NF-κappaB ligand (RANKL) in human periodontal fibroblasts and lymphocytes [30], [31]. RANKL is a key cytokine for bone resorption and could therefore be associated with the pathogenic mechanisms of periodontitis [32]. In addition, Cdt affects the oral epithelium *ex vivo* and therefore might contribute to impair the barrier function of this cell layer against invading microbes [33].

A functional Cdt toxin requires carriage of the three genes, *cdtA, cdtB,* and *cdtC* [27]. The genes are present in the majority of the *A. actinomycetemcomitans* strains that have been isolated, but the proportion of isolates that lack all or some of the genes varies among the populations studied [17], [22], [34]–[38]. The *cdt* genes reside in a genomic island of the variable region of the *A. actinomycetemcomitans* pangenome [7]. The activity of Cdt varies among different *A. actinomycetemcomitans* strains and an enhanced Cdt-activity (cell growth inhibition >65%) was equally distributed among serotype b and c strains [22], [36]. The presence of a specific immunoreactivity to Cdt has also been studied and assumed to be a marker for presence of Cdt-expressing *A. actinomycetemcomitans* [39], [40]. Interestingly, while all carriers of *A. actinomycetemcomitans* exhibit neutralizing antibodies to the leukotoxin, the systemic immunoreactivity to Cdt is not always capable to neutralize the toxin [40], [41]. Despite substantial evidence supporting that Cdt has an ability to function as a virulence factor in pathogens producing the toxin [42], the importance of Cdt in the pathogenesis of periodontal disease remains to be understood [9]. It is unknown if the toxic action of the Cdt may account for a part of the *A. actinomycetemcomitans*-associated periodontal disease process on-going in young populations, particularly of African descent [43]–[45]. Prior to this study, the occurrence of *A. actinomycetemcomitans* in West African adolescents and the potential role of the Cdt in relation to periodontal disease has never been undertaken.

The presence of *cdt*-encoding genes, Cdt-activity and its serotype distribution in *A. actinomycetemcomitans* isolated from Ghanaian adolescents were determined in the present study. A two-year prospective cohort study was undertaken to evaluate the carrier status and selected characteristics of *A. actinomycetemcomitans* in relation to progression of attachment loss (AL).

## Materials and Methods

### Subject Recruitment

**Figure 1:**
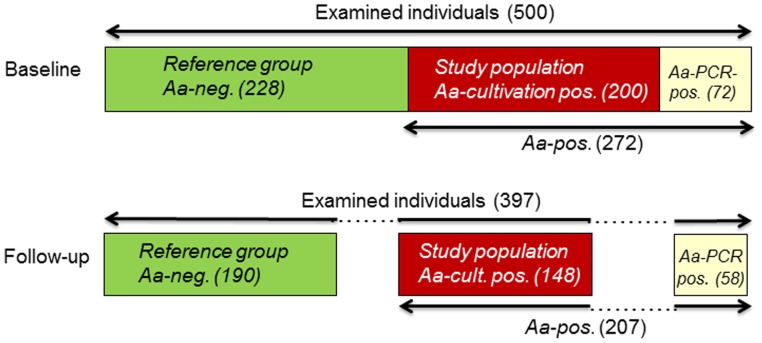
Flow chart overview of the study population and the detection of *A.* actinomycetemcomitans (*Aa*). At baseline, 500 adolescents were examined for the presence of *Aa* with cultivation and PCR-based methods. *Aa* was detected in 272 individuals and the bacterium could be isolated from 200 of them (*Aa* cultivation-positive group (red)). A second clinical examination (follow-up) was performed after 2 years and included 397 of the 500 individuals, 207 of the initially *Aa*-positive subjects and 148 of them from whom the bacterium had been isolated at baseline (red). The individuals without detectable *Aa* with any of the two methods used, were classified as negative for the bacterium (*Aa*-negative group (green)) and consisted of 228 individuals at the baseline examination and 190 at the follow-up examination. The number of individuals that were positive for *Aa* by PCR, but not with cultivation (yellow), was 72 at baseline and 58 of them were examined at the follow-up.

An adolescent West African population, described in details previously, was examined [45]. Briefly, a random cohort of 500 school children (mean age 13.2 years; SD ±1.5) was included in the cross-sectional study, which was performed in Accra, Ghana, in 2009 [45]. In the follow-up study, performed two years later, 397 (79.4%) of these individuals showed up for a periodontal re-examination (Fig. 1). The drop-out individuals consisted of children in families that moved to another area within the follow-up period or school children who dropped out from school. The school system had no information concerning the individuals that had left school.

Ethical clearance for the study was obtained from the Noguchi Memorial Institute for Medical Research, University of Ghana (IRB 000 1276), and from the local Ethical committee of Umeå University, Sweden (Dnr 2010-188-31M). Signed consents were received from the parents or the guardians of the children before they entered the study.

### Clinical Examination

All participants enrolled were given a full-mouth periodontal examination by the same, certified periodontist with identical procedures used at the baseline and at the follow-up examination [45]. Attachment loss (AL) was measured at the buccal aspect of the mesial and distal surfaces of all fully erupted permanent teeth, which gave a potential maximum of 56 sites per individual. AL was defined as the distance from the cemento-enamel junction (CEJ) to the bottom of the periodontal pocket or crevice and was calculated as the difference between two measurements (probing pocket depth and the distance from gingival margin to CEJ). Differences between baseline and follow-up were calculated at site level. The disease status was established using the cut-off point of AL ≥3 mm in one or more sites in the dentition. Individuals were defined as having progressive disease if they showed ≥ one site that had a progression of AL ≥3 mm based on data collected from the baseline and the follow-up examinations. None of the participants had received any periodontal treatment during the two-year follow-up period. Very few individuals had visited a dentist, except for treatment of pain related to the teeth. Traditional periodontal treatment strategies are not possible to introduce today in developing countries due to lack of dentists and resources for funding. Until affordable alternative strategies are available any form of periodontal treatment would be difficult to provide. This topic is a future challenge for the present project.

### Bacterial Isolates and Growth Conditions

Subgingival plaque samples from the 500 adolescents were collected for microbiological analysis, transported, and analyzed as described earlier [45]. In total, 792 *A. actinomycetemcomitans* isolates (1–7 per subject) were collected from 200 (40%) of the 500 subjects entered into the study at baseline. The leukotoxin promoter types (JP2/non-JP2) of isolates was determined by PCR technique [46], and the results are described in a previous study [45].

### Serotyping of *A. actinomycetemcomitans* by PCR

Suspensions of the strains were boiled for eight minutes and centrifuged. The supernatants were used as template when the 792 isolates were serotyped by PCR. The primers used in this study and the temperature profiles for amplification of the various genes have previously been described, for serotyping a–e [47], and for serotype f [17]. All isolates could be serotyped by the 6 primer pairs (a-f). No nonserotypable was found, making it irrelevant to consider testing for the recently described serotype g [18].

The PCR products were analysed by agarose (1.2%) gel electrophoresis in a Tris-acetate (40 mM; pH 8.3) buffer containing 1 mM EDTA. The gel was stained with ethidium bromide and photographed under ultraviolet light. Base pair sequences for each of the forward and reversed primers used and gel product size is presented in the supporting information (Table S1).

### *Cdt* Genotyping of *A. actinomycetemcomitans* by PCR

From the 792 serotyped and leukotoxin promoter-typed isolates, 249 isolates were selected for *cdt*-genotyping by PCR. One isolate from each of the 200 subjects were used, but when more than one serotype and/or leukotoxin promoter type (JP2 or non-JP2) were identified in the same subject, these additional isolates were included and summed up to 249. For the detection of the three Cdt genes (*cdtABC*), two PCR-based methods were used [36]. One of the methods was designed for detection of all three genes (*cdtABC*) and revealed a 2105 base pair (bp) product. When discrepancy between detection of *cdtABC* and Cdt-activity occurred, the individual *cdt* genes (*A, B* and *C*, respectively) were screened for by the second method [34]. The PCR products are documented as described for serotyping. Primer sequences, gene position, and gene fragment size for the *cdt* gene analyses, are shown in the supporting information (Table S1).

### Cdt-activity Test of *A. actinomycetemcomitans* in Cell Cultures Examined by Flow Cytometry

The 249 selected isolates were analyzed for Cdt-activity in a cell culture assay [14]. HL-60 cells (human carcinoma leukocyte cell line) were cultured in RPMI-1640 with 10% fetal bovine serum (Sigma-Aldrich). A suspension of OD^600 nm^ 2.0 was centrifuged (10 000×g, 10 min), and the supernatant added to the cultured cells. The HL-60 cells (1 ml 5×10^5^ cells/ml) were transferred to each well in a 24-well cell culture plate (Nunc) and mixed with 1 µl of each of the bacterial supernatants. After 24 h of incubation the cells were transferred to a 2 ml Eppendorf tube and washed with PBS by centrifugation (500×g, 5 min). The cell pellet was solved in 300 µl PBS and 900 µl ice-cold 99% ethanol and fixed for 1 h at 4°C. Cells were washed with PBS by centrifugation and treated with RNase (100 µl, 100 µg/ml, Sigma-Aldrich) for 15 min at 37°C. After the incubation, 400 µl of propidium iodide (Molecular Probes, Eugene, OR, USA) in 3.8 mM sodium citrate in PBS was added and further incubated in darkness for 1–3 h at 4°C. Cdt-activity was determined by the ability of the bacterial supernatants to inhibit proliferation and causing the typical accumulation of the target cells in the G2/M-phase examined and the increased cell size (FSC) by cell cycle analyses with flow cytometry (FACS Calibur, Becton Dikinson; Franklin Lakes, NJ, USA). Bacterial isolates that resulted in ≥50% of the target cell population in the G2/M-phase after 24 h incubation were classified as positive for Cdt-activity.

### Statistical Analysis

Data analyses were performed using SPSS 19.0 (SPSS Inc., Chicago, IL, USA) and STATA 8.0 (StataCorp LP., College Station, Texas, USA). In the statistical analyses, the primary outcome was progression of AL ≥3 mm in one or more sites at subject level, based on the collection of data performed at baseline (in November 2009) and at the follow-up (in November 2011). Descriptive statistics were performed using mean and standard deviation for the number of teeth with AL ≥3 mm per individual and group differences assessed using a non-parametric test (Mann-Whitney U test). The Mantel-Haenszel test was used for comparison of the distribution between groups of individuals harboring sites with AL ≥3 mm. The estimated risk associated with progression of AL ≥3 mm during a two-year follow-up period according to the carrier status of *A*. *actinomycetemcomitans*, characteristics as serotype and Cdt-activity (negative or positive), was evaluated by calculations of odds ratios (OR). A value of *p*<0.05 was considered statistically significant. The calculation of OR was repeated after exclusion of JP2-positive individuals, who were defined as carriers of the JP2-genotype based on positive plaque samples by the cultivation technique and/or by the PCR.

## Results

*A. actinomycetemcomitans* was isolated from 200 (40%) of the examined individuals at baseline. From these subjects, a collection of 249 isolates was selected, one isolate from each of the 200 subjects and additional isolates included from subjects where more than one serotype or more than one leukotoxin promoter type could be detected in the same sample. The highly leukotoxic JP2 genotype of *A. actinomycetemcomitans* was found in isolates from five (2.5%) of the individuals. All five of these individuals carried also the non-JP2 genotypes.

### Study Population and Subject Recruitment

Demographic characteristics from the baseline and the follow-up examination of the studied individuals are shown in Table 1. From the initially 500 examined individuals at baseline, 397 of them (79.4%) could be identified and were available for a two-year clinical follow-up examination (Fig. 1). Descriptive data for the number of individuals with affected sites with AL ≥3 mm and the number of affected teeth (mean) with AL ≥3 mm for the examined individuals, the selected study population of *A. actinomycetemcomitans* cultivation-positive individuals, and the reference group of individuals tested negative for the presence of this bacterium, are shown in Table 2. The mean age of the *A. actinomycetemcomitans*-positive individuals at baseline (n = 200) and at follow-up (n = 148) was 13.4 (SD; ±1.48) and 15.2 (SD; ±1.38), respectively, and for the *A. actinomycetemcomitans*-negative referents at baseline (n = 228) and at follow up (n = 190) the corresponding ages were 12.9 (SD; ±1.43) and 14.7 (SD; ±1.28), respectively. The odds ratios (OR) for having one or more sites with AL ≥3 mm was higher in the *A. actinomycetemcomitans*-positive group in relation to the *A. actinomycetemcomitans*-negative reference group at baseline and at follow up (OR = 2.197; 95% CI [1.355–3.561] *p = *0.001 and OR = 5.138; 95% CI [3.167–8.334] *p*<0.001) (Mantel-Haenszel test) (Table 2). Also the number of affected teeth (mean) with AL ≥3 mm was significantly higher among the *A. actinomycetemcomitans*-positive individuals than among the *A. actinomycetemcomitans*-negative reference group at both baseline (*p*≤0.001) and at the follow-up (*p*≤0.001) (Mann-Whitney U test) (Table 2). None of the examined individuals was cigarette smokers or had diabetes.

**Table 1:** Descriptive and demographic characteristics of the examined individuals at baseline and at the two-year follow-up examination.

Study population	Baseline (n = 500)		Follow-up (n = 397)	
Demographics				
Age (years)	mean	SD	mean	SD
	13.2	1.53	15.0	1.39
Gender	n	%	n	%
Male	232	46.4	176	44.3
Female	268	53.6	221	55.7

SD; standard deviation.

**Table 2:** Descriptive and clinical characteristics of the examined individuals (all), the study population (*Aa*-cultivation positive), and the reference group (*Aa*-negative) at baseline (BL), and at the two-year follow-up (FU) examination.

	Examined individuals			
Clinical	BL n = 500		FU n = 397	
	n	%	n	%
N individuals (%) with sites AL ≥3 mm	107	21.4	156	39.2
	mean	SD	mean	SD
N of teeth (mean) with AL ≥3 mm	0.52	1.37	1.92	3.34
	***Aa*** **-cultivation-positive**			
	BL n = 200		FU n = 148	
	**n**	**%**	**n**	**%**
N individuals (%) with sites AL ≥3 mm	54	27.0	82	54.4
	mean	SD	mean	SD
N of teeth (mean) with AL ≥3 mm	0.67	1.44	3.01	3.90
	**Reference group (** ***Aa*** **-negative)**			
	BL n = 228		FU n = 190	
	n	%	n	%
N individuals (%) with sites AL ≥3 mm	33	14.4	37	19.4
	mean	SD	mean	SD
N of teeth (mean) with AL ≥3 mm	0.34	1.28	0.89	2.63

AL; attachment loss; BL, at baseline; FU, at two-year follow-up.

*Aa; Aggregatibacter actinomycetemcomitans*.

SD; standard deviation.

### Serotyping

**Figure 2:**
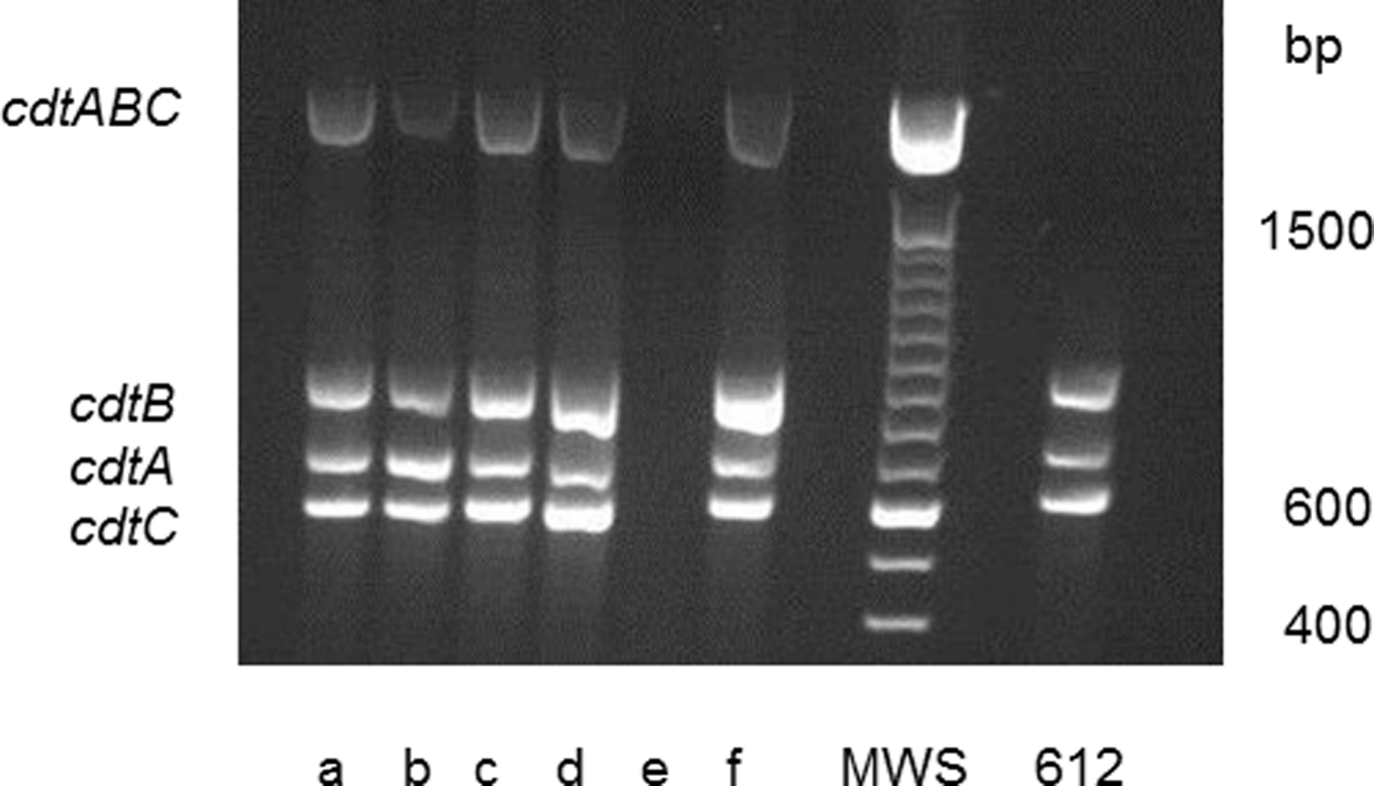
Agarose gel electrophoresis of PCR products of amplified cdt-genes in serotypes a through f isolates of *A.* *actinomycetemcomitans*, including a serotype a isolate (612), which was negative for detection of the *cdtABC* cluster, but positive for detection of the individual cdt genes. Size of the cdt gene cluster *cdtABC*, *cdtA*, *cdtB*, *cdtC*: 2105, 693, 862, 592 bp (base pair), respectively.

Serotyping of the 249 *A. actinomycetemcomitans* isolates showed presence of six different serotypes (a–f) (Fig. 2). Serotype c was the most frequently found and was detected in 104 (42.0%) of the isolates, while the less frequently found serotype e could be detected in only 8 (3.2%) of the isolates. The frequency of serotype a, b, d, and f in the examined isolates was 59 (23.7%), 47 (18.9%), 12 (4.8%), and 19 (7.6%), respectively. More than one serotype could be detected in samples from 45 (22.5%) of the 200 subjects. Only three individuals (1.5%) were poly-infected with three or more serotypes.

### *cdt*-genotype and Cdt-activity

**Figure 3:**
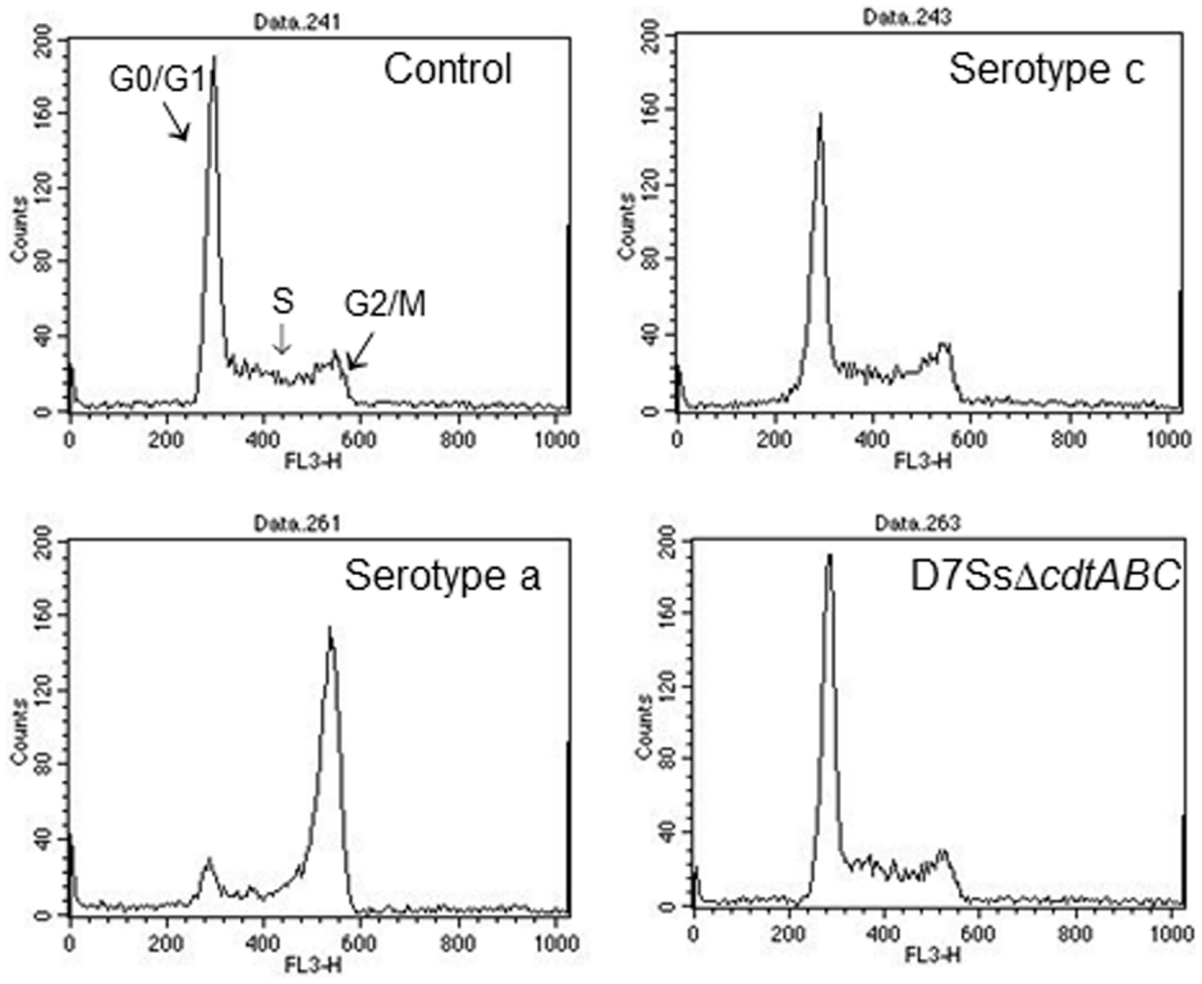
Histogram from flow cytometric analyses of leukocytes (HL-60 cells) exposed for 24 h to different extracts from various isolates of *A.* *actinomycetemcomitans*. The DNA-content of each exposed HL-60 cell were determined with PI-staining and indicate cell cycle phase of the analysed cell. The upper left panel (control) was exposed for 0.1% isoton NaCl, the upper right panel for 0.1% NaCl extract from an isolate with non-complete *cdt-*genome, the lower left panel for 0.1% NaCl extract from an isolate with intact *cdt-*genome and the lower right panel exposed to 0.1% NaCl extract from a *cdt* knockout mutant strain (D7SSΔ*cdtABC*).

**Figure 4:**
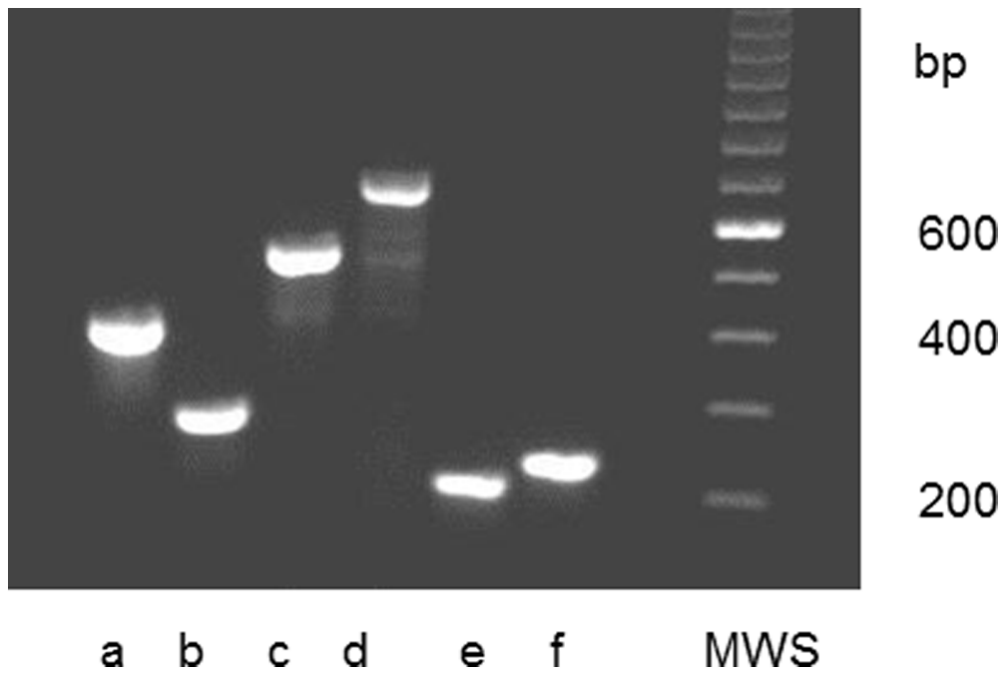
Agarose gel electrophoresis of PCR products amplified from serotypes a through f strains of *A.* *actinomycetemcomitans* isolated from a young Ghanaian population. Size of the serotype–associated gel fragments (a through f, bp (base pair): 428, 298, 559, 690, 211, 231.

**Figure 5:**
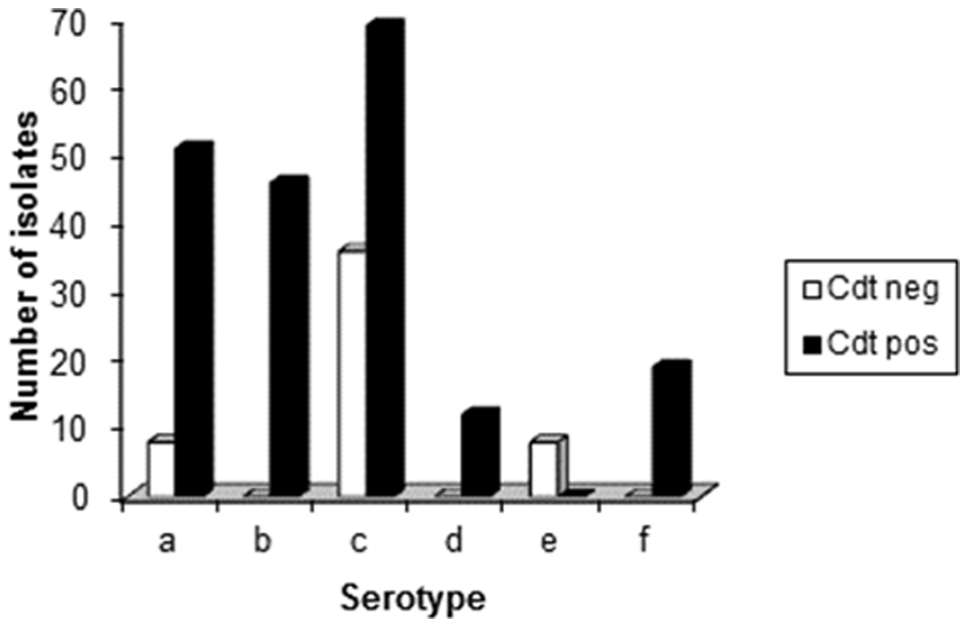
Distribution of isolates with Cdt-activity among the various serotypes of *A.* *actinomycetemcomitans*.

The activity of Cdt was examined in a cell culture-based assay by its ability to induce an accumulation of enlarged cells in the G2/M phase of growth (Fig. 3). Cdt-activity was detected in 196 (79%) of the 249 examined isolates. These 196 isolates with Cdt-activity also harboured all three *cdt*-genes (*A, B* and *C*) when examined by PCR and afterwards visualized in an agarose gel (Fig. 4). However, in one isolate the *cdt*-genes were found only by the method used for detection of the individual three *cdt*-genes. It may be possible that the method for amplifying the *cdt*-genes as a complete 2100 base pair product was not optimal for this isolate or, e.g. that a single base mutation had occurred resulting in unsuccessful annealing of the primers (amplification of the genes). The distribution of isolates with Cdt-activity and presence of all three *cdt*-encoding genes varied among the different serotypes of *A. actinomycetemcomitans.* All serotype b (n = 47), d (n = 12), and f (n = 19) isolates were positive for Cdt-activity and the *cdt*-genes, while all serotype e isolates (n = 8) lacked Cdt-activity and intact *cdt*-encoding genes (Fig. 5). A total of 35 (33.7%) of the serotype c isolates were tested negative for Cdt-activity, and for serotype a the number of negative isolates was 8 (13.6%). Taken together, these results showed a significant serotype-dependent pattern of *cdt*-genotypes in *A. actinomycetemcomitans* isolated from this population (Fig. 5). Isolates with the presence of all three *cdt*-encoding genes and a substantial Cdt-activity were considered as Cdt-positive, while the other isolates were counted as Cdt-negative.

### Carrier Status of *A. actinomycetemcomitans* and Progression of AL

The progression of AL ≥3 mm at one or more sites over a two-year follow-up period was examined in relation to the carrier status of *A. actinomycetemcomitans*. There was a significantly increased progression of AL in the *A. actinomycetemcomitans*-positive individuals (OR = 5.126, 95% CI [2.994–8.779] *p*<0.001) in relation to that of the *A. actinomycetemcomitans*-negative reference individuals (Table 3). Exclusions of individuals with presence of the JP2-genotype, detected by cultivation and/or PCR, resulted in for the *A. actinomycetemcomitans* positive individuals (n = 130) an OR of 4.323 (95% CI [2.482–7.530], *p*≤0.001).

**Table 3:** Presence of *A. actinomyctemcomitans* (*Aa*) with different serotypes, *cdt*-genotype and disease progression.

	OR	95% CI	p-value	Total number	Individuals (%) with progression of AL ≥3 mm
***Aa*** **-pos**	5.126	2.994–8.779	<0.001	148	63 (42.6)
*Aa*-serotype a	6.917	2.869–16.673	<0.001	26	13 (50.0)
*Aa*-serotype b	7.685	2.835–20.830	<0.001	19	10 (52.6)
*Aa*-serotype c	3.365	1.659–6.826	<0.001	55	18 (32.7)
*Aa*-Cdt-pos	5.237	3.000–9.143	<0.001	123	53 (43.1)
*Aa*-Cdt-neg	4.611	1.861–11.426	= 0.001	25	10 (40.0)
***Aa*** **-neg** *	1.000	reference	–	190	24 (12.6)

Odds ratio (OR) showing the association of progression of attachment loss (AL) ≥3 mm after a two-year follow-up period in relation to the carrier status of *Aa*.

OR, odds ratio; CI, confidence interval; AL, attachment loss. Significance (*p*<0.05).

* The reference group. Reference is the progression of AL in individuals that were *Aa*-negative.

### Serotype and Progression of AL

The most prevalent serotypes of *A. actinomycetemcomitans* isolated from the present study population were a, b, and c. In order to examine for the importance of the serotype of the bacterium for the progression of AL ≥3 mm, the individuals tested positive for one of these three serotypes were selected from the study population. In each group of individuals positive for any of the three selected serotypes, the OR for progression of AL compared to that of the *A. actinomycetemcomitans-*negative referents was high (Table 3). The highest OR for progression was associated with presence by the b serotype (OR = 7.685; 95% CI [2.835–20.830], *p*<0.001), closely followed by the a serotype (OR = 6.917; 95% CI [2.869–16.673], *p*<0.001), while the OR for progression was lower, however still significant, in the individuals positive for the c serotype (OR = 3.365; 95% CI [1.659–6.826], *p*<0.001). Due to the low number of individuals carrying *A. actinomycetemcomitans* of serotypes d, e, and f, these were not included in the analyses. The corresponding results for serotypes b and c after exclusion for the presence of the JP2-genotype strains were calculated. We also had to consider some individuals positive for serotype c as these were co-infected with serotype b JP2-genotype strains. For the individuals with the presence of the b serotype *A. actinomycetemcomitans* (n = 16), the OR was 6.917 (95% CI [2.374–20.152], *p*≤0.001), and for the individuals with the c serotype (n = 50), the OR was 2.964 (95% CI [1.413–6.219], *p* = 0.004). None of the individuals with the presence of the a serotype of the bacterium were in addition co-infected with the JP2-genotype.

### *cdt*-genotype and Progression of AL

Presence of Cdt-negative *A. actinomycetemcomitans*, as well as the presence of Cdt-positive bacteria showed a significant association with an increased progression of AL compared with that of the *A. actinomycetemcomitans*-negative referents (Table 3). The results for the *A. actinomycetemcomitans*-positive individuals (n = 148), for the individuals (n = 123) with Cdt-positive bacteria, and for the individuals (n = 25) with Cdt-negative bacteria were OR = 5.126 (95% CI [2.994–8.779], *p*≤0.001); OR = 5.237 (95% CI [3.000–9.143], *p*≤0.001), and OR = 4.611 (95% CI [1.861–11.426], *p* = 0.001), respectively. The OR after exclusion of individuals with presence of JP2 genotype bacteria was OR = 4.402 (95% CI [2.472–7.837], *p*≤0.001) for individuals (n = 108) with Cdt-positive bacteria and OR = 3.952 (95% CI [1.501–10.409], *p* = 0.005) for individuals (n = 22) with Cdt-negative bacteria.

## Discussion

In the present study, we have examined a collection of *A. actinomycetemcomitans* isolated from individuals included as a part of a prospective cohort study carried out in Accra, Ghana [45]. The proportion of individuals (79.4%) identified for the follow-up examination after two years was considerably high in the population chosen for this study, represented at baseline by a group of 500 medically healthy adolescents. This is the first microbiological study of *A. actinomycetemcomitans* from West-Africa, and neither *cdt*-genotype and activity nor serotypes have been reported on before based on such a collection of West-African *A. actinomycetemcomitans* strains.

Six different serotypes (a-e) were detected in the collection of 249 isolates from the 200 Ghanaian individuals. In line with earlier studies, examining isolates from Asian or American populations, the serotype c was the most prevalent (42.0%), followed by serotype a (23.7%), and serotype b (18.9%) [22]–[24]. The other three detected serotypes d, e and f were present in relatively low proportions being 4.8%, 7.6%, and 3.2%, respectively. This is also in accordance with previous studies [16], [21], [23].

The distribution of *cdt*-genotypes among the analysed isolates showed a serotype-dependent pattern that was unique for the present collection in relation to results from previous studies [19]–[24]. The present collection of *A. actinomycetemcomitans* is from a representative adolescent population consisting of otherwise healthy subjects, all living in the same geographic area located in West-Africa. Most comparable studies have dealt with isolates from patient cohorts with a history of periodontal disease [16], [19]–[23].

Cdt-activity was detected in 79% in the collection of the 249 selected isolates from 200 individuals. Previous studies, aimed at examining the expression of Cdt, has mainly focused on the presence of *cdt*-genes in bacteria isolated from periodontally diseased individuals [22], [23], [36], [40]. The results from these previous studies showed a substantial variation in the proportion of isolates that contained all three *cdt*-encoding genes. This may be explained by the different geographic origins and periodontal status of the examined individuals, but can also involve differences in the methodology for the detection of the *cdt*-genes. Two studies based on PCR-data from plaque samples have indicated a relatively low proportion of detectable *cdt*-genes in the *A. actinomycetemcomitans*-positive sites [21], [35]. However, variations in the methodology may explain the relatively low detection frequency of *cdt*-genes in these studies. Further, the *cdt*-genes are located in the variable region of the *A. actinomycetemcomitans* pangenome, which can explain the variations in the detection frequency of these genes in *A. actinomycetemcomitans* isolated from individuals of various origin [7]. Results from the present study showed that all isolates with Cdt-activity contained the three *cdt*-genes, while all the isolates without Cdt-activity lacked some of the *cdt*-encoding genes.

In the present study, we applied a cell culture-based method for determination of Cdt-activity that quantified cell cycle phase of the Cdt-exposed cells [14]. This method is more specific than the other commonly used method that demonstrates a general inhibition of the cell proliferation or effects on cell morphology and viability [22], [36], [38]. Examination of the accumulation of cells in the G2/M phase of the cell cycle is a specific marker for Cdt-intoxication and therefore a relevant marker for detection of Cdt-activity [13]. The complete correlation between the presence of an intact *cdt*-genome and a substantial activity of the toxin might depend on the specific methodology selected for the Cdt-activity analyses.

Despite substantial evidence supporting that Cdt has an ability to function as a true virulence factor in many pathogens producing the toxin [27], [42], the importance of Cdt in the pathogenesis of periodontal disease remains to be evaluated [9]. In our study, the presence of *A. actinomycetemcomitans* was significantly associated (p≤0.001) with progression of AL over a two-year observation period. This is in line with previous longitudinal studies examining the relation between the presence of *A. actinomycetemcomitans* and progression of AL in adolescent individuals [43], [48], [49]. In the present study, both individuals colonized with Cdt-positive or Cdt-negative bacteria were significantly more often included among the subjects that showed progression of AL (≥ 3 mm at one or more periodontal site) than the *A. actinomycetemcomitans*-negative individuals. As particularly the JP2 genotype has been strongly associated with progression of AL, it was important for us to eliminate this genotype from the analyses and afterwards repeat the data analyses. Therefore, exclusion of the JP2 genotype-positive individuals from the study population was done, but this did not change the results substantially. Thus, data suggests that the expression of Cdt might be of low importance for the pathogenesis of periodontitis, although further studies are needed to clarify whether this toxin can act and how to as a true virulence factor.

In the present cohort of Ghanaian adolescents, the progression of AL was examined in relation to the presence of serotype a, b, or c of *A. actinomycetemcomitans*. Using the progression of AL in the *A. actinomycetemcomitans*-negative individuals as the reference group, the presence of each of these three serotypes showed a significant association with progression of AL. Exclusion of the JP2-genotype positive individuals did not substantially change the estimates. Finding that the serotype b strains have the strongest relation to disease progression (OR = 7.685), this is in line with previous studies indicating an increased virulence in *A. actinomycetemcomitans* from serotype b [25], [26]. Although, the presence of *A. actinomycetemcomitans* as an important etiological factor was studied in this cohort of Ghanaian adolescents, an effect of other periodontal pathogens present concomitantly in the subgingival biofilm cannot be excluded.

In conclusion, the distribution of serotypes, *cdt*-genotypes and Cdt-activity of *A. actinomycetemcomitans* isolated from Ghanaian adolescents, showed a pattern that was comparable with results found in other populations. Progression of AL is mainly associated with the presence of *A. actinomycetemcomitans* and appears weakly associated with the *cdt-*genotype. All serotypes of *A. actinomycetemcomitans* studied were related to the progression of AL, serotype b showing the strongest association with disease progression.

## Supporting Information

Table S1 **Primer sequences, gene position, and gene fragment size for the serotype and** ***cdt*** **-genotype PCR analyses.**
(DOC)
