# Supplementary material for: Cytolethal Distending Toxin in Isolates of Aggregatibacter actinomycetemcomitans from Ghanaian Adolescents and Association with Serotype and Disease Progression
Source: PLoS One. 2013 Jun 14;8(6):e65781. doi: 10.1371/journal.pone.0065781 (PMC3683020; doi:10.1371/journal.pone.0065781)
Supplement: Table S1 — Primer sequences, gene position, and gene fragment size for the serotype and cdt -genotype PCR analyses. (DOC) [file pone.0065781.s001.doc]

Table S1. Primer sequences, gene position, and gene fragment size for the serotype and *cdt*-genotype PCR analyses.

| Primer | Oligonucleotide sequence | Gene position |
| --- | --- | --- |
| **Serotype a** | F 5’-GCAATGATGTATTGTCTTCTTTTGGA-3´ | 10554-10579 |
|  | R 5’-CTTCAGTTGAATGGGGATTGACTAAAAC-3´ | 10982-10955 |
| **Serotype b** | F 5’-CGGAAATGGAATGCTTGC-3´ | 15279-15296 |
|  | R 5’-CTGAGG AAGCCTAGCAAT-3´ | 15576-15559 |
| **Serotype c** | F 5’-AATGACTGCTGTCGGAGT-3´ | 1632381- 1632364 |
|  | R 5’-CGCTGAAGGTAATGTCAG-3´ | 1631833-1631850 |
| **Serotype d** | F 5’-TTACCAGGTGTCTAGTCGGA-3´ | 7742-7761 |
|  | R 5’-GGCTCCTGACAACATTGGAT-3´ | 8432-8413 |
| **Serotype e** | F 5’-CGTAAGCAGAAGAATAGTAAACGA-3´ | 16432-16454 |
|  | R 5’-AATAACGATGGCACATCAGACTTT-3´ | 16643-16620 |
| **Serotype f** | F 5’-AAAATTTCTCATCGGGAATG-3´ | 4813-4832 |
|  | R 5’-CCTTTATCAATCCAGACAGC-3´ | 5044 -5025 |
| ***cdt*ABC** | F 5’-ATGAAAAAGTTTTTACCTGGTC-3´ | 668-689 |
|  | R 5’-TTAGCTACCCTGATTTCTCC-3` | 2773-2754 |
| ***cdt*A** | F 5’-GGGGGCTAGTGGAGGATCTAAGGAGAGATATAATG-3’ | 497-523 |
|  | R 5’-GGGGGAGCTCTTAATTACCCTGTTGCTTCTAATACAG-3’ | 1190-1163 |
| ***cdt*B** | F 5’-GGGGGAATTCTAAGGAGTTTATAGCAATGGGTAAAG-3’ | 1196-1223 |
|  | R 5’-GGGGGGAATTCTTAGCGATCACGAACAAAACTAACAG-3’ | 2058-2032 |
| ***cdt*C** | F 5’-GGGGGGAATTCTAGTTTTGTTCGTGATCGCTAAGGAG-3’ | 2034-2059 |
|  | R 5’-GGGGGACTAGTTAGCTACCCTGATTTCTTCGCACCG-3’ | 2626-2603 |

Primer Gel fragment size

Serotype a 428 bp

Serotype b 297 bp

Serotype c 531 bp

Serotype d 690 bp

Serotype e 211 bp

Serotype f 231 bp

*cdtABC* 2105 bp

*cdtA* 693 bp

*cdtB* 862 b*p*

*cdtC* 592 bp
